# Cytoglobin in Hepatic Stellate Cells Plays Anti-Fibrotic Role in Chronic Liver Injury

**DOI:** 10.3390/antiox15030383

**Published:** 2026-03-19

**Authors:** Norifumi Kawada

**Affiliations:** Departments of Homeostatic Regulation and Liver Cancer Treatment, Graduate School of Medicine, Osaka Metropolitan University, Osaka 545-8585, Japan; kawadanori@omu.ac.jp

**Keywords:** cytoglobin, hepatic stellate cells, liver fibrosis, liver cancer, oxidative stress

## Abstract

Cytoglobin (Cygb) was discovered in 2001 as a cytoplasmic globin predominantly expressed in hepatic stellate cells (HSCs). While its initial physiological role remained elusive, subsequent studies using Cygb-deficient mouse models of liver injury have demonstrated that Cygb exerts protective effects against liver fibrosis and inflammation. It achieves this by regulating HSC activation, thereby preserving hepatic homeostasis. Furthermore, accumulating evidence suggests a significant role for Cygb in hepatocarcinogenesis. Analysis of human liver tissues and cell-based models has further confirmed the critical involvement of CYGB in liver pathology. Functionally, Cygb acts as an antioxidant protein that mitigates oxidative stress, a property that appears to modulate transforming growth factor-beta signaling and downstream fibrogenic responses. Based on these findings, therapeutic strategies employing recombinant CYGB for the treatment of human liver cirrhosis are currently being explored, and their potential clinical applications are eagerly anticipated.

## 1. Introduction

Liver disease has emerged as a major global health challenge. In 2020 alone, approximately 900,000 deaths were attributed to liver disease, with an additional 830,000 resulting from liver cancer [1]. The primary etiologies of liver cancer exhibit significant regional variations: hepatitis B virus infection predominates in East Asia, alcohol consumption is the leading cause in Eastern and Central Europe, and hepatitis C virus infection is most prevalent in Western Europe, the United States, and Japan [2]. Notably, recent years have seen a rapid increase in liver cancer cases associated with metabolic dysfunction-associated steatotic liver disease (MASLD), driven by the global rise in obesity and type 2 diabetes [3,4].

Regardless of the underlying cause, liver cancer typically develops within the context of chronic liver disease or cirrhotic liver tissue [5]. The cyclic process of hepatocyte necrosis and regeneration promotes genetic mutations and the methylation of tumor suppressor genes, such as *p53*, leading to progressive DNA damage [6]. Furthermore, tissue fibrosis, angiogenesis, and a suppressive immune environment collectively establish a microenvironment that facilitates the survival and proliferation of cancer cells [7]. Given this pathological landscape, the early identification and elimination of causative factors, alongside the “purification” of liver tissue through the induction of “de-fibrosis,” are critical strategies for cancer prevention [8]. Consequently, research into the pathogenesis of liver fibrosis has advanced significantly.

In this article, I review the progress in our molecular and cellular understanding of liver fibrosis and discuss the pivotal role of cytoglobin (Cygb)—expressed in hepatic stellate cells (HSCs)—in the fibrotic process, based on the current literature.

## 2. Anatomy of the Liver

The liver is a substantial organ situated in the upper abdomen, typically weighing between 1.3 and 1.5 kg in adults. A defining characteristic of the liver is its dual blood supply from the hepatic artery and the portal vein. The portal vein, in particular, originates from the digestive tract and spleen, delivering venous blood enriched with dietary nutrients, gut-derived substances, ingested drugs, and immune cells. This vessel serves as a critical conduit for transporting materials to hepatocytes, the primary functional units of the liver. Upon entering the liver, the portal vein branches and intertwines with the terminal branches of the hepatic artery to form specialized capillaries known as sinusoids. The sinusoidal walls are composed of liver sinusoidal endothelial cells (LSECs). The luminal side is primarily inhabited by Kupffer cells (phagocytic macrophages) and pit cells (which exhibit natural killer cell activity), while the exterior is surrounded by HSCs, which are liver-specific pericytes. Hepatocyte cords are organized in an orderly fashion adjacent to these sinusoids, and the intervening gap is termed the space of Disse [9,10].

As cytoglobin was subsequently discovered within HSCs, a brief historical overview of these cells is warranted. In 1876, the German pathologist Professor Carl von Kupffer identified cells containing “inclusion bodies” between hepatocytes and sinusoids using gold staining, naming them *Sternzellen* (star cells). However, definitive evidence of their existence remained scarce for decades. In 1951, Professor Toshio Ito, an anatomist at Gunma University in Japan, observed cells distinct from hepatocytes that stained red with Sudan III in human liver tissue; he termed these “fat-storing cells” [11], which later became known as Ito cells. It was Professor Kenjiro Wake of Tokyo Medical and Dental University who rigorously replicated these historical experiments. Utilizing the Golgi silver method, he elucidated the three-dimensional morphology of the cell body, proving that Kupffer’s *Sternzellen* and Ito’s fat-storing cells were identical [12]. Furthermore, Professor Wake demonstrated through fluorescence microscopy that the lipid droplets identified by Professor Ito were, in fact, vitamin A [13]. While these cells were briefly referred to as “lipocytes” in the United States and other regions [14], the nomenclature was later standardized to “hepatic stellate cells” to avoid confusion, following a proposal by Dr. Scott Friedman [15].

## 3. Involvement of HSCs in Fibrotic Process of the Liver

In the 1970s, Professor Hans Popper and colleagues at the Mount Sinai School of Medicine observed that pathologically and anatomically fibrotic liver tissues contained lipocytes, which are specialized cells for Vitamin A storage [14]. In 1985, Scott L. Friedman demonstrated through primary cell culture experiments that the cells responsible for producing extracellular matrix (ECM) components, such as type I and III collagen, were not hepatocytes but HSCs adhering to plastic dishes [16]. This finding, combined with the development of methods for isolating highly purified HSCs [17], confirmed Friedman’s hypothesis and catalyzed numerous subsequent discoveries.

It is now established that factors such as transforming growth factor-beta (TGF-β) [18], platelet-derived growth factor [19], and reactive oxygen species (ROS) activate HSCs. Upon activation, HSCs express α-smooth muscle actin (αSMA) and exhibit increased contractility through the production of endothelin-1 and angiotensin II [20]. Furthermore, activated HSCs themselves become a potent source of cytokines, including chemokines and interleukins (IL-1 and IL-6) [21]. Beyond their role in fibrosis, activated HSCs are recognized as critical components of the liver cancer microenvironment [22]. Recent studies have also shown that senescent HSCs induce the senescence-associated secretory phenotype (SASP) [23], among other complex roles in liver pathology [24,25].

Based on this evidence, various inhibitors have been developed under the hypothesis that modulating HSC activation could provide effective anti-fibrotic therapies. However, while several candidates have demonstrated efficacy in animal models, none have yet been confirmed as effective in human clinical trials [26].

## 4. Discovery of Cytoglobin from Rat HSCs

HSCs isolated from rats, mice, and humans are known to undergo spontaneous activation when cultured on plastic dishes—a phenomenon widely utilized in experimental models. Around 2000, we sought to identify proteins specifically expressed in rat HSCs by separating HSC-derived proteins using two-dimensional electrophoresis and subsequently analyzing peptide fragments from individual spots via mass spectrometry [27]. During this investigation, we identified two peptide fragments (PGDMER(I/L)ER and ANCEDVGVA) derived from a 21 kDa protein with an isoelectric point (pI) of 6, which were not registered in the Swiss-Prot or GenBank databases at that time. Based on the sequence information from these two fragments, the protein was cloned and characterized as a heme-containing protein consisting of 190 amino acids. We initially named this protein stellate cell activation-associated protein [28].

Subsequent cloning of human cytoglobin (CYGB) revealed that Cygb shares 40% amino acid sequence homology with myoglobin, while human CYGB exhibits 95% homology with its rat and mouse orthologs [29]. In 2002, Trent III JT and colleagues identified a hemoglobin family member termed histoglobin, which is expressed across diverse human tissues. This globin shares less than 30% sequence identity with other human hemoglobins and functions as a hexacoordinate hemoglobin with ligand-binding characteristics distinct from those of neuroglobin [30]. Concurrently, Burmester et al. identified a 20.9 kDa protein, also named CYGB, by analyzing globin proteins within expressed sequence tag databases [31].

Functionally, Cygb exhibits a myoglobin-like role in oxygen (O_2_) metabolism by facilitating diffusion to the mitochondria. It also acts as a sensor, participating in signal transduction pathways that modulate regulatory protein activity in response to fluctuations in concentration. The backbone structure of the Cygb monomer displays a traditional globin fold characterized by a three-over-three helical sandwich, a helical arrangement essentially conserved across all globins [32,33]. Furthermore, Cygb protects cells from oxidative stress by neutralizing reactive species such as nitric oxide (NO) and hydrogen peroxide (H_2_O_2_). It also possesses the ability to oxidize lipids, generating signaling molecules that may regulate cellular responses to damage [34,35].

Neuroglobin (Ngb), another member of the globin family discovered in 2000, is predominantly expressed in the brain [36]. Both Cygb and Ngb are hexacoordinated globins, meaning the iron atom in the heme group is bound by two internal histidine side chains. This configuration requires a structural shift to accommodate external ligands; consequently, they exhibit a significantly higher affinity for O_2_ than hemoglobin, allowing them to retain oxygen even under severely hypoxic conditions [37,38]. Unlike the ubiquitously expressed Cygb, Ngb is primarily localized to the central and peripheral nervous systems and the retina [39]. Structurally, Cygb can form dimers via disulfide bridges between cysteine residues, whereas Ngb remains primarily a monomer. Similar to Cygb, Ngb facilitates O_2_ diffusion to the mitochondria in neurons, thereby playing a critical role in preventing neuronal death [40].

## 5. Role of Cytoglobin in Liver Injury, Fibrosis and Carcinogenesis

### 5.1. Oxidative Stress and Liver Injury

Oxidative stress serves as a pivotal driver in the progression of liver diseases, functioning as a critical bridge between initial metabolic insults and chronic organ failure. This pathological state originates from a fundamental imbalance where the production of ROS overwhelms the endogenous antioxidant capacity of hepatocytes. Within the hepatocyte, mitochondrial dysfunction and endoplasmic reticulum stress constitute the primary intracellular sources of ROS [41,42].

Excessive levels of O_2_^−^ and H_2_O_2_ initiate lipid peroxidation, specifically targeting polyunsaturated fatty acids within cellular membranes. This oxidative degradation generates highly reactive electrophilic byproducts, such as 4-hydroxynonenal and malondialdehyde, which are known to form DNA adducts and potently trigger apoptosis via the activation of the c-Jun N-terminal kinase (JNK) signaling pathway [43].

The subsequent transition from cellular damage to systemic inflammation is marked by the release of Damage-Associated Molecular Patterns (DAMPs) from dying hepatocytes [44]. These molecular signals activate resident macrophages, or Kupffer cells, prompting the secretion of pro-inflammatory cytokines, including tumor necrosis factor-alpha (TNF-α) and IL-1β [45]. This inflammatory milieu further exacerbates ROS production, establishing a self-perpetuating vicious cycle of necroinflammation.

The critical transition to hepatic fibrosis is mediated by the activation of HSCs. Under physiological conditions, HSCs exist in a quiescent state, primarily serving as storage sites for Vitamin A. However, persistent stimulation by ROS and TGF-β triggers their transdifferentiation into myofibroblast-like cells, which are characterized by excessive extracellular matrix deposition [46,47].

### 5.2. Role of Cygb in Liver Trauma

Regarding its biochemical functions, Cygb is known to bind both O_2_ and NO and possesses peroxidase and catalase activities—common functional hallmarks of heme proteins [30,31,32]. In 2006, Xu et al. utilized a modified recombinant adeno-associated virus-2 to overexpress full-length rat Cygb, demonstrating that its overexpression suppressed the activation of primary cultured rat HSCs and conferred protection against liver injury and fibrosis induced by carbon tetrachloride (CCl_4_) or bile duct ligation (BDL) [48]. Given that Cygb is predominantly expressed in HSCs, we sought to elucidate its physiological role in the liver, particularly in the context of fibrosis, by generating Cygb knockout (KO) mice. Long-term observation of these Cygb KO mice revealed that elderly mice developed spontaneous liver inflammation and fibrosis without external stimuli. These pathological changes were accompanied by the expression of αSMA, an HSC activation marker, and ultimately progressed to hepatocellular carcinoma (HCC) [49]. In HSCs isolated from these KO mice, we observed significantly elevated levels of αSMA and collagen 1α1, as well as pro-inflammatory cytokines (IL-1, IL-6, and TNF-α) and chemokines. Furthermore, enhanced superoxide (O_2_^−^) production, detected via DHE assay, indicated that these cells had transitioned into a senescence-associated secretory phenotype (SASP)-like state.

In a diethylnitrosamine (DEN)-induced liver carcinogenesis model, no tumors were observed in wild-type (WT) mice after 36 weeks of 0.05 ppm DEN administration; in striking contrast, 48% of Cygb KO mice developed cancer, demonstrating that Cygb deficiency markedly predisposes mice to malignancy [50]. Similar results were obtained in a choline-deficient L-amino acid-defined diet (CDAA) model: while WT mice developed steatohepatitis after 32 weeks, 100% of Cygb KO mice developed liver cancer. In the liver tissues of these KO mice, HSC activation, fibrosis, and inflammatory responses were significantly exacerbated, alongside increased oxidative DNA damage in hepatocytes, as evidenced by elevated γH2AX expression [51]. Furthermore, in a cholestatic liver fibrosis model induced by BDL, liver injury and fibrosis were significantly more severe in KO mice than in WT controls [52]. Collectively, these findings suggest that Cygb deficiency exacerbates liver injury, promotes tissue oxidative stress and HSC activation, intensifies inflammation and fibrosis, and ultimately facilitates hepatocarcinogenesis.

Subsequently, we generated Cygb-mCherry transgenic (Tg) mice, which harbor 10 copies of the *Cygb* gene and express mCherry under the control of the native *Cygb* promoter. In these Tg mice, liver fibrosis was significantly suppressed in BDL, thioacetamide (TAA), and CDAA models. HSCs isolated from these Tg mice exhibited suppressed mRNA expression of αSma and collagen 1α1. These results suggest that overexpression of Cygb inhibits HSC activation and suppresses liver fibrosis [53].

Cygb serves as a dynamic antioxidant, oxygen sensor, and regulator of NO homeostasis [54]. Cygb acts as a potent NO dioxygenase (NOD), converting NO into nitrate under normoxic conditions [54,55]. In the absence of Cygb, NO levels rise significantly, leading to nitrosative stress [56]. While Cygb is found in HSCs, its absence directly impacts neighboring hepatocytes. Excess NO diffuses into hepatocytes and reversibly inhibits cytochrome c oxidase, a critical enzyme in the mitochondrial electron transport chain [56]. This inhibition suppresses respiratory function and triggers the production of ROS. Cygb provides essential protection against oxidative and nitrosative DNA damage [57]. By scavenging ROS and regulating NO, it prevents lipid peroxidation and oxidative DNA strand breaks that can lead to genomic instability and hepatocarcinogenesis [55,56].

## 6. Cytoglobin Gene Expression Regulation

In mammals, the regulation of the *CYGB* gene is a multi-layered process involving genetic, epigenetic, and environmental factors.

### 6.1. Transcriptional Regulation

The *CYGB* promoter contains several key binding sites for transcription factors that allow the cell to respond to physiological stress:

• Hypoxia-Inducible Factor 1 (HIF-1): Although its responsiveness is less pronounced than that of erythropoietin, the *CYGB* promoter contains Hypoxia Response Elements. Under hypoxic conditions, HIF-1 binds to these sites to upregulate expression, potentially protecting cells from hypoxia-induced ROS [58].
• TLR2-SAPK/JNK Pathway: Recent reports have demonstrated the induction of ***CYGB*** through the Toll-like receptor (TLR) 2-mediated stress-activated protein kinase/Jun-terminal kinase (SAPK/JNK) pathway in human HSCs [59].

### 6.2. Epigenetic Regulation

Cytoglobin is frequently cited as a tumor suppressor gene, and its regulation via DNA methylation is a critical area of study:

• CpG Island Methylation: The *CYGB* promoter features a dense CpG island. In various malignancies, including lung, esophageal, and head and neck cancers, this region often becomes hypermethylated, effectively silencing the gene. This loss of expression is thought to promote tumor growth by impairing the cell’s capacity to manage oxidative DNA damage [60,61].

### 6.3. Response to Oxidative Stress and Fibrosis

The most distinct regulatory feature of cytoglobin is its dramatic upregulation during fibroproliferative diseases:

• Radical Scavenger Function: When fibroblasts transition into myofibroblasts (the primary effectors of scarring), *CYGB* expression spikes. This likely serves as a protective mechanism to buffer the high levels of ROS generated during collagen synthesis [62,63].
• NO Levels: Cytoglobin can function as an NO dioxygenase. Elevated levels of NO or its derivatives can influence signaling pathways that provide feedback into *CYGB* transcription [64,65].

### 6.4. Regulation Comparison: CYGB vs. Hb/Mb

The regulatory logic of CYGB is fundamentally different from that of hemoglobin (Hb) and Myoglobin (Mb). To understand why CYGB is unique, it helps to compare its regulation to the “classic” globins. While Hb and Mb are primarily regulated by metabolic demand and development, CYGB is regulated as a cellular stress sentinel [31,66,67] (Table 1).

**Table 1:** Comparison between CYGB and classical Hb/Md.

Feature	Hemoglobin (Hb)	Myoglobin (Mb)	Cytoglobin (CYGB)
Tissue Specificity	Erythrocytes only	Striated muscle (Heart/Skeletal)	Ubiquitous (esp. Fibroblasts/Neurons)
Key Regulator	Erythropoietin (EPO) and Iron	Locomotor activity and Calcium	Oxidative Stress and Fibrosis
Promoter Control	Lineage-specific enhancers	MEF2, NFAT (Muscle-specific)	HIF-1, AP-1, Sp1 (Stress-responsive)
Binding State	Pentacoordinate (Easy O_2_ swap)	Pentacoordinate (Storage)	Hexacoordinate (Likely signaling)

### 6.5. Regulation of CYGB Expression by Fibroblast Growth Factor 2 (FGF2) and Lawson

Using HHSteC cells, a human HSC cell line, fibroblast growth factor 2 (FGF2) was shown to induce CYGB transcription. At the same time, FGF2 suppressed αSMA expression in HHSteCs. The mechanism involved FGF2-induced phosphorylation of JNK and c-JUN, and the JNK inhibitor PS600125 inhibited CYGB transcription. Chromatin immunoprecipitation revealed that binding of phosphorylated c-JUN to a consensus motif (5-TGA(C/G)TCA), located 218 to 222 bases from the transcription initiation site, in the CYGB promoter triggered transcription [68]. Recent studies have shown that lapachol (a 1,4-naphtoquinone analog) and its analog, lawsone, which were identified through screening for inhibitors of the human COL1A2 promoter, suppress HSC activation while simultaneously enhancing the expression of CYGB. The effect of lawsone on COL1A1 and αSMA expression in HSCs is related to the role of YAP [69].

## 7. Potential for Recombinant Human Cytoglobin as a Protein Therapy

**Figure 1:**
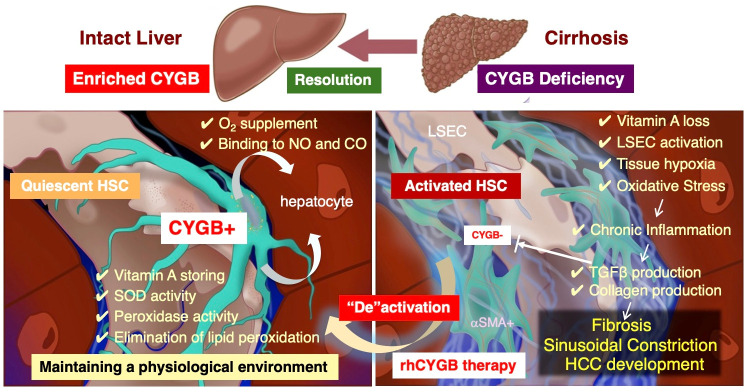
Therapeutic potential of human recombinant CYGB in hepatic fibrosis. In the healthy human liver, Cytoglobin (CYGB) is primarily localized within hepatic stellate cells (HSCs), where it plays a vital role in maintaining an antioxidant and oxygen-rich perisinusoidal environment. However, as chronic hepatitis progresses toward fibrosis and eventual cirrhosis, the expression of CYGB is significantly suppressed by transforming growth factor beta (TGF-β). This loss of CYGB renders HSCs increasingly vulnerable to oxidative stress, locking them into a persistently activated state. This pathological activation not only accelerates the deposition of fibrous tissue but also induces DNA damage in hepatocytes, creating a microenvironment conducive to liver cancer development. Recombinant human CYGB therapy aims to break this cycle by restoring CYGB expression within these activated HSCs. By replenishing this essential protein, the therapy facilitates the deactivation of HSCs, ultimately promoting the regression of fibrosis and the recovery of hepatic function. αSMA, alpha-smooth muscle actin; CO, carbon monoxide; CYGB, cytoglobin; HCC, hepatocellular carcinoma; HSC, hepatic stellate cell; LSEC, liver sinusoidal endothelial cell; NO, nitric oxide; O_2_, oxygen; rh, recombinant human; SOD, superoxide dismutase; TGF-β, transforming growth factor beta.

Recent studies suggest that CYGB may function beyond its role as a gas-binding protein, possessing significant potential as a therapeutic agent. Specifically, recombinant human cytoglobin (rhCYGB) has emerged as a promising “protein therapy” capable of reversing liver fibrosis and atherosclerosis, at least in preclinical animal models (Figure 1).

### 7.1. Attenuation of Liver Injury and Fibrosis by rhCYGB Administration

Dong et al. first reported the protective effects of subcutaneous rhCYGB (2 mg/kg) against CCl_4_-induced hepatic injury and fibrosis, noting a significant upregulation of antioxidant defenses [70]. Furthermore, the same research group established that rhCYGB (3 mg/kg) mitigates alcohol-induced liver injury by enhancing antioxidant capacity [71]. They subsequently identified relevant serum proteins to further elucidate the underlying therapeutic mechanisms [72,73].

### 7.2. Direct Deactivation of HSCs

In the fibrotic liver, HSCs undergo activation and progressively secrete ECM components, such as collagen, primarily in response to TGF-β signaling. Experimental evidence indicates that the exogenous addition of rhCYGB to culture media suppresses the expression of αSMA and collagen in a dose- and time-dependent manner, suggesting that rhCYGB directly inhibits HSC activation [74]. Upon intravenous administration, rhCYGB is selectively internalized by activated HSCs via clathrin-mediated endocytosis. Once intracellular, rhCYGB functions as a potent scavenger of ROS, thereby promoting the deactivation of myofibroblasts or, in some instances, inducing apoptosis [74].

### 7.3. Induction of Interferon-Beta (IFN-β)

Recent studies have further elucidated that the therapeutic role of rhCYGB extends beyond its primary antioxidant function; it also modulates specific immune signaling pathways. Notably, rhCYGB triggers the autocrine secretion of interferon-beta (IFN-β) from HSCs. It is known that the production of Type I interferon including IFN-β is related to the activation of TANK-binding kinase 1 (TBK1) [75]. In fact, it was confirmed that the addition of rhCYGB causes phosphorylation of TBK1 in human HSCs. As a potent anti-fibrotic cytokine, IFN-β plays a critical role in downregulating the transcription of key fibrogenic genes, including *COL1A1* and the contractile protein αSMA, which collectively contribute to hepatic stiffness [74].

## 8. The Role of CYGB in Human Carcinogenesis

Liver fibrosis and its progression to cirrhosis frequently serve as a predisposition for HCC. Consequently, understanding the regulatory role of CYGB is critical for elucidating the mechanisms of tumor development both within the liver and in extrahepatic tissues. A hallmark finding in CYGB oncology is its frequent transcriptional silencing via promoter hypermethylation. This epigenetic inactivation has been documented across various malignancies—including lung, esophageal, head and neck, and breast cancers—where diminished CYGB expression often correlates with tumor aggressiveness and poor clinical prognosis.

### 8.1. Hepatocellular Carcinoma (HCC)

In the hepatic microenvironment, CYGB is predominantly expressed in HSCs [28]. The loss of CYGB impairs the scavenging of ROS, precipitating a state of chronic oxidative stress that activates inflammatory macrophages and accelerates the fibrotic cascade [49,50,51]. Beyond its role in oxidative homeostasis, CYGB serves as a molecular “brake” on oncogenic signaling; specifically, it has been shown to inhibit the AKT/ERK1/2/Cyclin D1 signaling axis [76]. The loss of CYGB expression removes this inhibitory control, thereby promoting uncontrolled cellular proliferation and contributing to the poor survival outcomes observed in patients with HCC [50,51].

### 8.2. Extrahepatic Malignancies

The tumor-suppressive role of CYGB extends to various other organs through distinct epigenetic and molecular mechanisms:

• **Esophageal Cancer:** The CYGB gene, located on chromosome 17q25, undergoes promoter methylation very early during the malignant transformation of esophageal cells [77].
• **Head and Neck Squamous Cell Carcinoma:** In clinical samples, CYGB mRNA expression levels show a significant positive correlation with hypoxia markers (e.g., HIF-1A) and a marked negative correlation with promoter methylation, suggesting its role in the hypoxic tumor microenvironment [61].
• **Non-Small-Cell Lung Cancer:** Frequent silencing by hypermethylation has positioned CYGB as a potential biomarker for early detection in sputum samples [78].
• **Pancreatic Ductal Adenocarcinoma (PDAC):** Interestingly, elevated CYGB expression is primarily localized within the carcinoma cells themselves [79] [. Low CYGB expression is significantly associated with shorter disease-free and disease-specific survival. Furthermore, CYGB expression inversely correlates with several pro-oncogenic factors, including Phosphoinositide 3-kinase, p-AKT, IL-6, and vascular endothelial growth factor. Multivariate analysis has confirmed that CYGB expression serves as an independent prognostic factor alongside clinical stage in PDAC. These clinical findings are supported by in vivo data, where Cygb overexpression was shown to suppress 7,12-dimethylbenzanthracene-induced pancreatic tumorigenesis in mouse models [78]. The fact that Cytoglobin-expressing cells in the pancreas are pancreatic stellate cells is highly intriguing, given their functional parallels with those in the liver [80,81].

## 9. Future Perspectives

The discovery of Cygb and the subsequent elucidation of its multifaceted roles in hepatic pathophysiology have opened new avenues for liver research. While substantial progress has been made in understanding its function as a ROS scavenger and a regulator of HSC activation, several critical questions remain to be addressed to translate these findings into clinical practice.

First, the precise molecular mechanisms by which Cygb is selectively internalized by activated HSCs through clathrin-mediated endocytosis warrant further investigation. Identifying the specific receptors or membrane proteins involved in this process could facilitate the development of targeted drug delivery systems, minimizing off-target effects in other tissues. Furthermore, while the induction of IFN-β by rhCYGB represents a paradigm shift in our understanding of globin-mediated immune modulation, the signaling crosstalk between Cygb-induced cytokines and the broader immune microenvironment in the fibrotic liver remains largely unexplored [72].

Second, the transition from preclinical animal models to human clinical trials remains the most significant challenge. Although rhCYGB has shown remarkable efficacy in reversing fibrosis and preventing hepatocarcinogenesis in rodents, the long-term safety, optimal dosage, and pharmacokinetics in humans must be rigorously evaluated. Given the global rise in MASLD, exploring the therapeutic potential of rhCYGB in the context of metabolic syndrome and insulin resistance will be a priority.

Finally, Cygb’s role as a tumor suppressor suggests that it could serve as a valuable biomarker for early cancer detection or as a prognostic indicator for patients with chronic liver disease. Future research should focus on large-scale clinical cohorts to correlate *CYGB* expression levels or promoter methylation status with disease progression and therapeutic outcomes.

In conclusion, cytoglobin is no longer viewed merely as a passive oxygen-binding protein but as a dynamic player in hepatic homeostasis and repair. Harnessing the therapeutic potential of this unique globin may eventually provide a breakthrough in the treatment of liver cirrhosis and the prevention of liver cancer, offering hope to millions of patients worldwide.

## Data Availability

No new data were created or analyzed in this study. Data sharing is not applicable to this article.

## Abbreviations

Abbreviations are detailed in the text at their first appearance and listed here:

αSMA	α-smooth muscle actin
BDL	bile duct ligation
CCl_4_	carbon tetrachloride
CDAA	choline-deficient L-amino acid-defined diet
Cygb	cytoglobin
DEN	diethylnitrosamine
ECM	extracellular matrix
FGF2	fibroblast growth factor 2
Hb	hemoglobin
HSCs	hepatic stellate cells
HCC	hepatocellular carcinoma
HIF-1	hypoxia-inducible factor 1
IFN-β	interferon-beta
ILs	interleukins
JNK	c-Jun N-terminal kinase
KO	knockout
LSEC	liver sinusoidal endothelial cells
MAFLD	metabolic dysfunction-associated fatty liver disease
Mb	myoglobin
Ngb	neuroglobin
NO	nitric oxide
NOD	nitric oxide dioxygenase
O_2_	oxygen
PDAC	pancreatic ductal adenocarcinoma
ROS	reactive oxygen species
Rh	recombinant human
SASP	senescence-associated secretory phenotype
SAPK/JNK	stress-activated protein kinase/jun-terminal kinase
O_2_^−^	superoxide
TLR2	toll-like receptor
TBK1	TANK-binding kinase 1
TAA	thioacetamide
TGF-β	transforming growth factor-beta
Tg	transgenic
TNF-α	tumor necrosis factor-alpha
WT	wild-type
